# Electrophysiological Recording With Activated Iridium Oxide: Effective Electrode Area, Not Total Impedance, Determines Performance

**DOI:** 10.1111/ejn.70640

**Published:** 2026-07-20

**Authors:** Alexander R. Harris, Ben J. Allitt, Antonio G. Paolini

**Affiliations:** ^1^ Department of Biomedical Engineering University of Melbourne Melbourne Victoria Australia; ^2^ Higher Education College Chisholm Institute Dandenong Victoria Australia; ^3^ ISN Psychology Institute for Social Neuroscience Ivanhoe Victoria Australia; ^4^ School of Psychology and Public Health La Trobe University Bundoora Victoria Australia

**Keywords:** electroanalysis, electrophysiology, iridium oxide, neural implant, neural recording

## Abstract

Platinum electrodes are used in many bionic devices; however, they have limited charge injection capacity (CIC) and signal‐to‐noise ratio (SNR), restricting performance. Iridium oxide has substantially larger CIC and reduced impedance compared to platinum. While these types of electrochemical measurements are typically used to infer improved electrophysiological performance, there are no studies demonstrating this correlation. This article compares the electrochemical and acute electrophysiological recording performance of iridium and electrochemically activated IrOx. Increased iridium activation reduced total impedance at low‐intermediate frequencies and steady‐state electroactive area but had no impact on linear diffusion electroactive area. The total impedance had a poor correlation with background noise, SNR and spike count. The results contribute to the previous literature indicating linear diffusion electroactive area determines the number of neurons within recording distance of the electrode, impacting SNR and spike count. Measurements of impedance are mainly of benefit as an indirect measure of linear diffusion electroactive area, in which case the measurement and analysis must be performed appropriately. A wide range of IrOx activation states can be produced with increased CIC, without concern of neural recording performance impacts. However, higher oxidation states resulting in electrode dissolution or increase in electrode resistance may limit IrOx chronic performance.

## Graphical abstract

Linear diffusion electroactive area determines the number of neurons within recording distance of an implanted electrode, impacting signal‐to‐noise ratio and spike count. Impedance measurements are mainly of benefit as an indirect measure of electrode area.

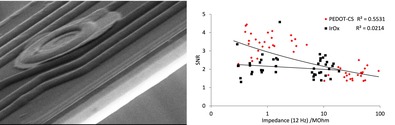

**Abbreviations**
CIC: charge injection capacity
CPE: constant phase element
EIS: electrochemical impedance spectroscopy
IC: inferior colliculus
IrOx: iridium oxide
PEDOT‐CS: chondroitin sulfate doped poly(3,4‐ethylenedioxythiophene)
R: resistor
RMS: root mean square
SNR: signal‐to‐noise ratio
Wo1: finite‐length Warburg diffusion impedance

## 1 Introduction

Electrodes can be used to record and stimulate electrically excitable cells (Cogan 2008). Electrophysiological recordings can be used to understand and classify normal tissue function and the impact of disease or trauma (Stieglitz 2019). The output from these recordings can subsequently be used to control prosthetic devices (Flesher et al. 2021) or for monitoring disordered states allowing patient interventions (Stirling et al. 2021). Electrophysiological stimulation can be used to drive sensory percepts or control tissue function when normal function has been affected by disease or trauma (Clark 2009; Wagner et al. 2018; Goyal et al. 2021).

The electrodes used for bionic applications must be highly conductive, biocompatible and biostable, with platinum used for many clinical devices. However, platinum is a relatively hard material with limited charge injection capacity (Harris et al. 2018a, 2018b). In order to deliver sufficient charge without corroding the electrode or inducing water electrolysis, platinum electrodes must also be relatively large. This can lead to stimulation of a large volume of tissue resulting in off‐target side effects (Harris 2020). Larger electrode size also increases the overall implant size, which enhances the likelihood of glial scar formation around the electrodes, resulting in changes to tissue composition and structure (McConnell et al. 2009), larger electrode‐target tissue distance and a decrease in electrophysiological performance (Rousche and Normann 1998; Wellman et al. 2018).

The limited charge injection capacity of platinum has generated significant interest in the development of novel materials for bionic applications (Harris and Wallace 2018). Iridium oxide has been of particular interest, as iridium metal can be activated to multiple oxidation states resulting in very large charge injection capacities. While iridium oxide can be fabricated by sputter coating, it is easier to create and control the formation of iridium oxide by electrochemical pulsing of an iridium electrode in an electrolyte (Kang and Shay 1983; Beebe and Rose 1988). There are numerous publications using activated iridium for electrophysiological stimulation studies (Agnew et al. 1986; McCreery et al. 1992; McCreery et al. 2006).

Electrodes used for electrophysiological recording require a low background noise and large signal‐to‐noise ratio (SNR). These properties are normally assessed by measuring the electrodes' impedance and typically reported at 1 kHz (despite the limited utility of this value) (Harris et al. 2019; Harris et al. 2025). Activation of iridium reduces the electrode impedance at low‐intermediate frequencies and would be expected to improve its electrophysiological recording performance (Negi et al. 2010). Yet there are very few studies that report the use of activated iridium for electrophysiological recording (McCreery et al. 2006; Liu et al. 1999; Joshi‐Imre et al. 2019). This may be due to a concern that variation in impedance with iridium oxidation state will impact on the electrodes' recording function.

To address this concern, this article builds on a previous collection of studies we have undertaken with Neuronexus electrode arrays assessing the relationship between electrochemical and acute electrophysiological recording performance utilising electrodes modified with a variety of doped conducting polymers (Harris et al. 2013; Harris, Molino, et al. 2014; Harris, Morgan, et al. 2014). This previous work has demonstrated that electrode coatings can alter the effective electrode area, charge storage capacity, charge injection capacity, impedance and electrophysiological performance including background noise and signal‐to‐noise ratio. However, all of these measurements have entailed concurrent increase in electrode area and signal‐to‐noise ratio with reduced total impedance and background noise. This has prevented a more detailed analysis of the relationship between each of these electrode parameters. This is of concern as total impedance is dependent on multiple parameters, and there are different measures of electrode area (Trasatti and Petrii 1992; Harris et al. 2018a). Therefore, this article utilises the same electrochemical and electrophysiological techniques as the previous studies of conducting polymer modified electrodes, but applies them to electrochemically activated iridium oxide electrodes. Iridium oxide is a particularly interesting material for these studies, as the relationship between effective electrode area and total impedance differ to doped conducting polymers (Harris et al. 2017; Harris and Paolini 2017). This article's novel results provide important new information on the relationship between electrode area and total impedance with electrophysiological recording performance, enabling rational development of next generation bionic devices.

## 2 Methods

### 2.1 Materials and Electrode Coating

**FIGURE 1:**
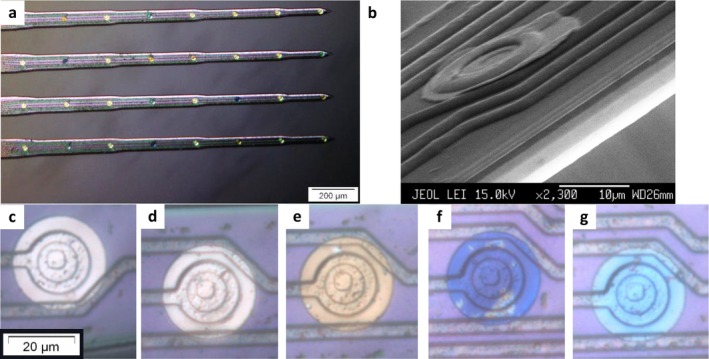
Optical and electron microscopy of iridium electrodes: (a) whole 32 channel array, (b) close‐up image of an electrode and connecting tracks and (c–g) after activation for 0, 50, 125, 250 and 500 pulses.

Hexaammineruthenium (III) chloride (Ru(NH_3_)_6_Cl_3_) and 99.0% di‐sodium phosphate (Fluka) were used as received. Electrode arrays had 4 shanks and 32 iridium electrodes (8 electrodes per shank) of 413 μm^2^ nominal geometric area and 200‐μm pitch (Neuronexus Technologies—A4x8‐5mm‐200‐200‐413). Electrodes were tested in a three‐electrode configuration on a CHI660B potentiostat with CHI684 multiplexer (CH Instruments) using an Ag/AgCl (3‐M KCl) as reference electrode and Pt wire as counter electrode. Individual microelectrodes were electrochemically activated in a solution of 0.3‐M phosphate buffer in deionised water as recommended by the electrode manufacturer. Activation was achieved by applying a cathodic pulse of −0.8 V for 0.5 s and then an anodic pulse of 0.8 V for 0.5 s for four different numbers of repetition cycles (50, 125, 250 and 500). Two probes were tested, four electrode sites at each number of activation cycles (Figure 1), leaving 16 unactivated iridium electrodes as controls.

Electrodes were imaged using a BX61 optical microscope (Olympus), and the geometric area was measured with ImageJ (Figure 1). Electrochemical analysis was undertaken in 0.3‐M phosphate buffer in deionised water, and the electroactive areas were measured by addition of 5‐mM Ru(NH_3_)_6_^3+^. Test solutions were not degassed. Cyclic voltammetry was performed over a range of 0.8 to −0.8 V versus Ag/AgCl at a scan rate of 100 mV s^−1^. Electroactive area measurements were undertaken over a range of 0 to −0.5 V varying the scan rate from 10 mV s^−1^ to 1 V s^−1^. Electrochemical impedance spectroscopy (EIS) was performed at 0 V with a 10‐mV amplitude over a frequency range of 10–100,000 Hz to compare with previous data on conducting polymer modified neural electrodes. Equivalent circuit fitting of the EIS data was performed with ZView.

### 2.2 In Vivo Testing

Experimental procedures were performed in a sound attenuating Faraday cage on an anti‐vibration table. Hooded Wistar rats weighing over 200 g were anesthetised with urethane (20% v/v in distilled water, 1.3 g/kg i.p., Sigma‐Aldrich). The animal was secured in a stereotaxic frame (David Kopf Instruments) fitted with a hollow ear bar in the left ear. Animal temperature was monitored continuously via a rectal probe and maintained at 37.5°C using an ATC1000 DC temperature controller (World Precision Instruments). A craniectomy was performed to access the right inferior colliculus (IC). An Ag/AgCl wire reference electrode wrapped in saline saturated cotton wool was placed into the dorsal region of the animal’s neck. The multichannel probe was then inserted at a 19° rostro‐caudal angle with reference to Lambda using stereotaxic coordinates and a rat brain atlas (Paxinos and Watson 2007) approximately 2 mm into the brain, towards the IC. White noise bursts were generated by a RX6 multifunction processor and PA5 programmable attenuator (Tucker‐Davis Technologies) controlled by custom software developed in OpenEx. Sound was delivered through the left ear bar using an EC1 electrostatic speaker driven using an ED1 electrostatic speaker driver (Tucker‐Davis Technologies). Prior to use, the speaker was calibrated by attaching the sound generation system to one end of the ear bar with a 1/8‐in. 4138‐A‐015 microphone and amplifier unit and 2829 4‐Channel Microphone Power Supply (Brüel and Kjær) coupled to the opposite end using a 3‐mm‐long rigid plastic tube to mimic the rat's ear canal. The electrode was then advanced into the IC using a motorised microdrive (Sutter Instruments), whilst monitoring the neural response via a PZ2 high impedance amplifier and RZ2 bioamp processor (Tucker‐Davis Technologies) with band‐pass filtering (300–5000 Hz), until roughly the bottom 3 electrodes on each shank displayed acoustically driven activity.

An acoustic stimulation protocol of 300 repetitions of 50 ms white noise bursts (rise‐fall time 10 ms, Gaussian distributed noise, 1–44 kHz) at a 1 s repetition rate was then delivered at 70 dB sound pressure level, while recording the multiunit activity at each electrode (acquired at a sampling rate of 24.4 kHz). On completion of the acoustic stimulation protocol, the probe was advanced ~200 μm into the IC so that each electrode was in approximately the same position as the more distal electrode from the first measurement. The acoustic stimulation was then repeated, and the probe advanced in 200 μm steps until all of the electrodes had recorded acoustically evoked activity. The probe was then retracted in 200 μm steps using the same acoustic stimulation protocol to determine the reproducibility of the measurements and potential damage caused from the probe insertion. After in vivo recording, the electrodes were carefully retracted from the animal and gently rinsed with deionised water before storing. All animal procedures were in accordance with the ‘Australian code for the care and use of animals for scientific purposes’ and approved by the RMIT University Animal Ethics Committee (AEC Number 1315) published previously (Harris, Morgan, et al. 2014).

### 2.3 Data Analysis

Data from acoustically evoked responses were imported into Matlab for offline analysis. A Fourier transform of the complete 300‐s noise pulse train was performed. A bandpass filter of 300–5000 Hz was applied for measuring multi‐unit activity. For each electrode site, the average of the bandpass filtered RMS measured during acoustic stimulation (RMS_stim_) for the complete 50‐ms stimulation period was averaged from the 300 repetitions at one electrode depth. To eliminate artefacts stemming from neuronal refractory periods, we calculated the average bandpass‐filtered root mean square (RMS_bkgd_) exclusively from the final 300 ms of the 950 ms window, outside the acoustic stimulation period. This value was then averaged over 300 repetitions at a single electrode depth. The SNR was calculated from (RMS_stim_/RMS_bkgd_) with the SNR classification taken from Kim et al. (2010) (where low SNR < 3.5, medium SNR 3.5–4.0 and good SNR > 4.0). A spike was measured where the recorded potential was > 4.2 × SD of the RMS from the previous 1 s with an exponential weighting of signal for recency. The ‘during’ and ‘outside’ acoustic stimulation spike count was then performed over the same time periods as above. The spike count difference was calculated from (spike count during stimulation) − (spike count outside stimulation). Each electrode site was classified as ‘in’ the IC when acoustically driven neural activity induced a spike count difference greater than 45% of the maximum recorded spike count difference over the whole experiment. Data for each electrode site was averaged across all electrode depths ‘in’ the IC to reduce error due to variations in biological noise (the number of recordable neurons in the vicinity of the electrodes).

## 3 Results and Discussion

### 3.1 Microscopy and Electrochemistry

Microelectrodes were activated with varying numbers of voltage pulses and imaged by optical microscopy. Unactivated iridium was bright silver; following activation, they displayed patches of brown after 50 pulses, were fully brown after 125 pulses, dark blue after 250 pulses and a pale blue after 500 pulses (Figure 1e–g). The silver colour is consistent with iridium metal, brown colouring being the formation of the poorly conductive Ir (OH)_3_, dark blue the more conductive IrO_2_ and the light blue being a mix of higher iridium oxidation states (Harris et al. 2017). There was no change in the electrodes' geometric area with iridium activation.

**FIGURE 2:**
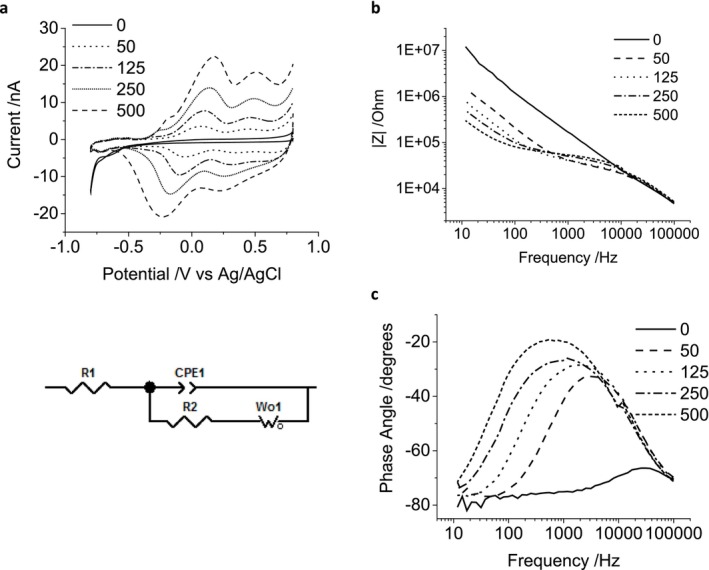
Electrochemical response after varying number of activation pulses in 0.3‐M Na_2_HPO_4_: (a) typical cyclic voltammetry at 100 mV s^−1^ and (b, c) electrochemical impedance spectroscopy at 0 and 10 mV amplitude with fitted equivalent circuit.

Cyclic voltammetry of the unactivated iridium was largely featureless, with a small reduction peak around −0.6 V most likely due to reduction of dissolved oxygen (Figure 2a). Following iridium activation with 50 pulses, reduction peaks appeared around 340, −57 and −720 mV with oxidation peaks at −593, 70 and 445 mV. There was some shift in the peak potentials and increase in peak magnitude with increased number of activation pulses. The faradaic processes visible in the voltammetry are associated with a series of redox reactions of the form ${\mathrm{IrO}}_{x}{\left(\mathrm{OH}\right)}_{y}+zH^{+}+ze^{-}⇌{\mathrm{IrO}}_{x-y}{\left(\mathrm{OH}\right)}_{y+z}$. Further discussion of the voltammetric response and charge density of the electrodes can be found in our previous publication (Harris et al. 2017).

EIS was performed at the defined and reproducible 0 V versus Ag/AgCl rather than the open circuit potential which varies with iridium oxidation. At 0 V, charge transfer on the unactivated iridium is largely associated with capacitance, while increasing levels of faradaic charge are available on activated iridium (Figure 2a). The impedance response of unactivated iridium displayed a decrease in total impedance versus frequency and near −90° phase angle, consistent with an RC equivalent circuit (Figure 2b,c). Following activation with 50 pulses, the total impedance at high frequencies was unchanged but decreased substantially at low‐intermediate frequencies. The phase angle also displayed a peak around 3 kHz, and the bandwidth or cut‐off frequency (where the phase angle is −45°) was ~810 Hz. With increasing number of activation pulses, the total impedance at intermediate frequencies (around 1 kHz) increased slightly, while decreasing at low frequencies (around 10 Hz). The peak in the phase angle also increased in magnitude and shifted to lower frequencies; the cut‐off frequency also shifted to lower frequencies. An equivalent circuit comprising resistors (R1 and R2), a constant phase element (CPE1), and finite‐length Warburg diffusion impedance (Wo1) provided a good fit of the activated iridium EIS. Further discussion of the impedance response and the equivalent circuit fitting has been demonstrated elsewhere (Harris and Paolini 2017).

The effective electrode area was assessed by the addition of Ru(NH_3_)_6_^3+^ to the test solution. Reduction of Ru(NH_3_)_6_^3+^ at fast voltammetric scan rates produced peak shaped voltammograms indicative of a linear diffusion profile to the unactivated electrode surface (Figure S1) (Harris et al. 2017). Measurement of the peak height (*i*_p_) can be used to calculate a linear diffusion effective area (*A*) according to

(1) $$i_{p}=\left(2.69\times 10^{5}\right)n^{3/2}{\mathrm{AD}}^{1/2}c\upsilon^{1/2}$$

where *n* is the number of electrons transferred, *D* is the diffusion coefficient (9.0 × 10^−6^ cm^2^ s^−1^) (Barsan et al. 2009), *c* is the concentration and *ν* is the scan rate. However, after activation, only quasi‐reversible voltammograms were obtained. This was most likely caused by slow electron transfer kinetics or increased electrode resistivity. Fitting of the response using DigiElch indicated the linear diffusion electroactive area was unaffected by the activation process. A roughness factor of ~1.2 could be calculated from the geometric and linear diffusion electroactive area (Harris et al. 2017).

**FIGURE 3:**
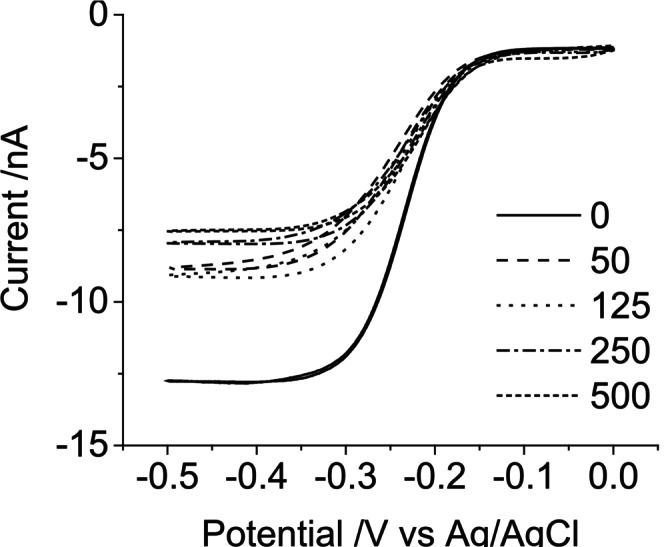
Typical background subtracted cyclic voltammetry at 10 mV s^−1^ of 5 mM Ru(NH_3_)_6_^3+^ in 0.3 M Na_2_HPO_4_ of iridium electrodes with varying numbers of after activation pulses.

At slow voltammetric scan rates, diffusion of Ru(NH_3_)_6_^3+^ to the microelectrode obtains a radial profile. This results in a sigmoidal shaped voltammetric response (Figure 3). The limiting current (*i*_ss_) at a disc electrode has the form:

(2) $$i_{\mathrm{ss}}=4\text{nFDcr}$$

where *F* is the Faraday constant and *r* is the electrode radius. On other electrode geometries, Equation (2) provides an equivalent disc radius. However, it was previously shown that the radial diffusion profile to the iridium Neuronexus electrodes is better modelled as a ring electrode due to the three‐dimensional structure of the electrode having a protruding ring with a recessed centre (Figure 1b) (Harris et al. 2017). The steady‐state current at a ring electrode is expressed as

(3) $$i_{\mathrm{ss}}=\text{nFDC}\frac{\pi^{2}\left(a+d\right)}{\ln16\left(a+d\right)/\left(d-a\right)}$$

where *a* is the inner radius and *d* the outer radius of the ring (Aoki 1993). A steady‐state response was seen on all electrodes, but the limiting current decreased in magnitude with increasing number of activation pulses. The gradient of the steady‐state curve also decreased with increasing number of activation pulses, indicating slow electron transfer kinetics or increased electrode resistivity. The decrease in electroactive area with electrode activation was attributed to iridium oxide (particularly Ir(OH)_3_) having a higher resistivity than iridium metal; a higher lateral resistivity may reduce the steady‐state effective electroactive area (Harris et al. 2017). Reduction of Ru(NH_3_)_6_^3+^ may then be restricted to the more conductive raised ring region of the electrode.

Electroanalysis has shown activation of iridium electrodes leads to a decrease in impedance at low‐intermediate frequencies, no change in linear diffusion electroactive area and a decrease in steady‐state diffusion electroactive area. In contrast, modification of neural electrodes with conducting polymers resulted in a decrease in impedance but with an increase in both measures of electroactive area (Harris et al. 2019; Harris et al. 2025; Harris et al. 2013). Subsequently, activated iridium electrodes provide great opportunity to interrogate the relationship between effective electrode area, impedance and electrophysiological performance.

### 3.2 Electrophysiology

**FIGURE 4:**
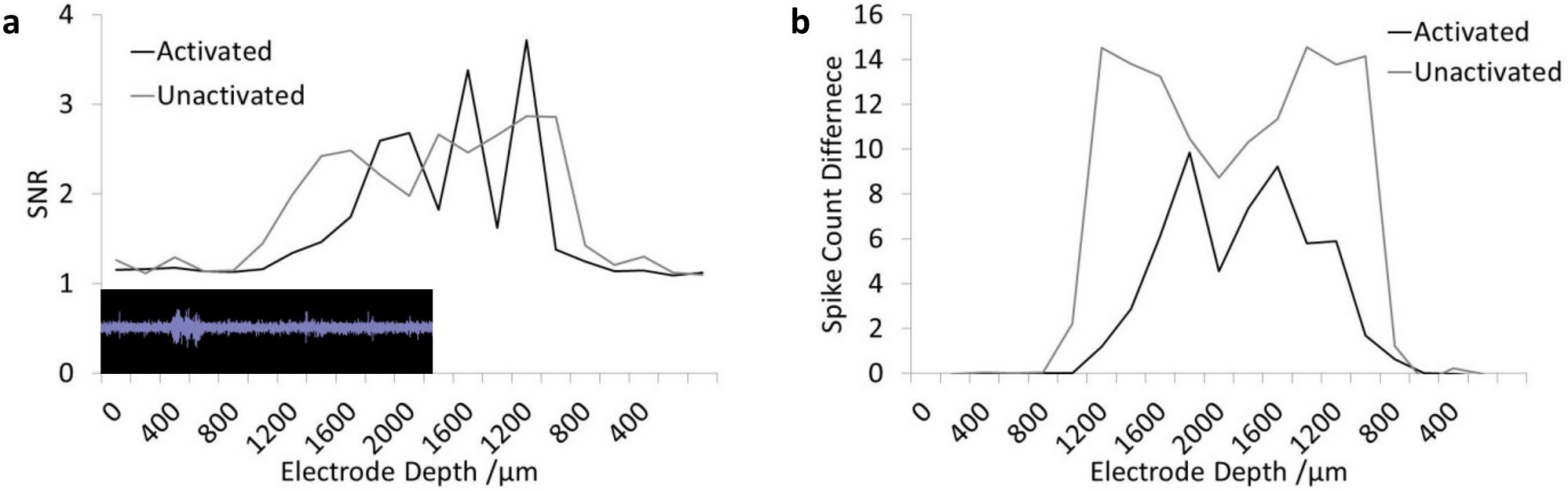
(a) Typical SNR and (b) spike count difference of an unactivated and activated (125 pulses) iridium electrode during insertion and withdrawal into the IC. (Inset) Typical streaming data showing RMS_bkgd_ of ~10 μV with increased RMS_stim_ during acoustic stimulation from a 50 ms white noise burst.

The electrophysiological response was monitored as the microelectrode arrays were inserted into the inferior colliculus (IC) of rats. Streaming data (inset Figure 4) produced an RMS_bkgd_ of ~10–20 μV due to spontaneous biological activity and thermal or electrochemical potential noise ($V_{\mathrm{rms}}^{\mathrm{th}}$) associated with fluctuations of charge carriers (electrons and ions) resulting in voltage fluctuations. White noise pulses were presented to the ear to induce multiunit activity, resulting in an increase in RMS during stimulation (RMS_stim_). When the most distal electrodes detected acoustically driven activity, the series of 300 noise pulses were presented. The probe was then inserted and retracted in 200 μm steps measuring the multiunit activity during acoustic pulses. The RMS_stim_ and SNR varied with position due to biological noise (variation in electrode‐neuron distance, number of neurons adjacent the electrode etc.) with no clear transition from outside to inside the IC (Figure 4a). In contrast, the spike count was close to 0 outside the IC during acoustic stimulation and rose sharply during acoustic stimulation when inside the IC (Figure 4b). A 45% maximum of spike count difference gave the most consistent measurement when used to define when an electrode was ‘in’ the IC.

The average RMS_bkgd_ of all electrodes in the IC was around 15 μV (Table 1). The RMS_bkgd_ magnitude was unaffected by electrode activation; however, its coefficient of variation decreased. The average RMS_stim_ in the IC was approximately 30 μV, resulting in a SNR of ~2 with a relatively uniform coefficient of variation across activation status. The maximum SNR at any position was classified as good (< 4.0) on five electrodes and medium (3.5–4.0) on four electrodes, with the remaining being low (< 3.5) (Kim et al. 2010). However, the maximum SNR was highly impacted by biological noise and was unreliable. The SNR corrected for biological noise was classified as low on all electrodes but was far more consistent. The spike count difference was ~10 and did not vary with electrode activation.

**TABLE 1:** Average, standard deviation and coefficient of variation of outside stimulation RMS potential, signal‐to‐noise ratio and mean during stimulation spike count measured from 64 electrodes on two electrode arrays in two separate animals.

Activation pulses	Outside stimulation RMS potential (μV)			Signal‐to‐noise ratio			Mean during stimulation spike count		
	Ave	SD	CV	Ave	SD	CV	Ave	SD	CV
0	16.7	10.6	0.64	2.06	0.44	0.21	10.2	4.2	0.41
50	11.3	1.4	0.12	2.71	0.96	0.35	12.3	5.7	0.46
125	14.0	2.3	0.17	2.23	0.39	0.17	10.9	5.6	0.51
250	13.0	1.3	0.10	2.03	0.28	0.14	9.4	4.0	0.42
500	14.1	4.3	0.30	2.11	0.80	0.38	9.0	6.5	0.72

There are links between the electrochemical properties of an electrode and its electrophysiological performance. The RMS_bkgd_ is impacted by thermal noise:

(4) $$V_{\mathrm{rms}}^{\mathrm{th}}=\sqrt{4k_{b}TZ^{\prime }\Delta f}$$

where *k*_*b*_ is Boltzmann's constant, *T* is the absolute temperature, *Z′* is the real part of the impedance and Δ*f* is the measuring bandwidth (Baranauskas et al. 2011). As an example, a 1 MΩ electrode used in the current setup would result in a thermal noise of ~9 μV. However, the reported noise measurements have been obtained in an animal, where other noise sources exist. Differences between this calculated value and the measured RMS_bkgd_ are most likely due to biological noise. In an RC circuit, the thermal noise equation simplifies to

(5) $$V_{\mathrm{rms}}^{\mathrm{th}}=\sqrt{\frac{k_{b}T}{C}}$$

More complex equivalent circuits will affect the thermal noise. A decrease in thermal noise will also increase the SNR and subsequently increase the number of spikes detected. Therefore, the impedance can have a correlation with RMS_bkgd_, SNR and spike count. However, it was previously shown with conducting polymer modified electrodes that the impedance must be measured at sufficiently low frequencies, where the electrode dominates the response (Harris et al. 2019; Harris et al. 2025).

**FIGURE 5:**
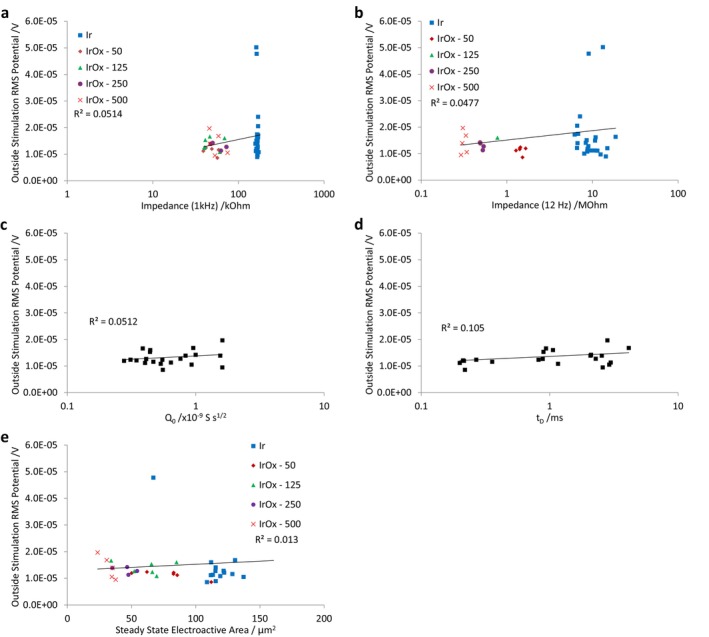
RMS_bkgd_ of unactivated and activated iridium electrodes versus (a) impedance at 1 kHz, (b) impedance at 12 Hz, (c) fitted admittance, (d) fitted diffusional time constant and (e) steady state diffusion electroactive area. The fitted trendlines are linear.

**FIGURE 6:**
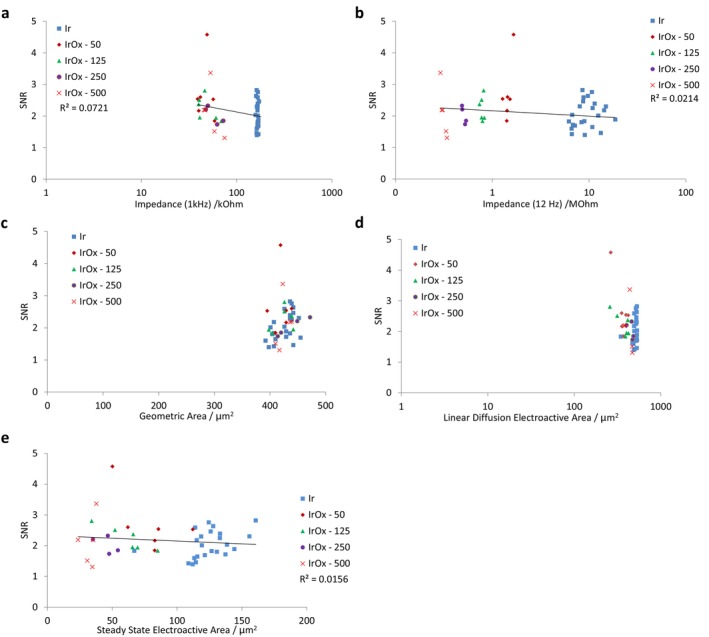
SNR of unactivated and activated iridium electrodes versus (a) impedance at 1 kHz, (b) impedance at 12 Hz, (c) geometric area, (d) linear diffusion electroactive area and (e) steady state diffusion electroactive area. The fitted trendlines are linear.

Plots of RMS_bkgd_ (Figure 5) and SNR (Figure 6) versus total impedance at 12 Hz, steady‐state electroactive area and the fitted equivalent circuit parameters of admittance and diffusional time constant (both of these fitted parameters had moderate‐strong correlations with number of activation pulses; Harris and Paolini 2017) displayed a separation of data points across the X‐axis according to the number of activation pulses. In contrast, plots versus impedance at 1 kHz, geometric and linear diffusion electroactive area were each clustered into a single group. Despite separation of data points with number of activation pulses on some plots, there were very low correlations between RMS_bkgd_ (Figure 5), SNR (Figure 6) and spike count difference (data not shown) against all electrochemical measurements. This is in stark contrast to the medium‐high correlation coefficients observed with conducting polymer modified electrodes (Harris et al. 2019; Harris et al. 2025). An overlay of SNR versus impedance at 12 Hz for activated iridium and a representative conducting polymer coated electrode can be seen in Figure S2.

The differences between activated iridium and conducting polymer coated Neuronexus electrodes can be more clearly demonstrated by comparing unmodified electrodes with individual coatings of similar impedance at 12 Hz (iridium activated by 500 pulses—318 kΩ [Harris and Paolini 2017]; and 45‐s deposition of chondroitin sulfate doped poly(3,4‐ethylenedioxythiophene) [PEDOT‐CS]—604 kΩ [Harris et al. 2016a]). Compared to unactivated iridium, activated iridium had no change in geometric or linear diffusion electrode area, a decrease in radial diffusion electrode area (27% of unactivated iridium), a decrease in total impedance at 1 kHz (36% of unactivated iridium) and 12 Hz (3% of unactivated iridium), yet **no** change in RMS_bkgd,_ SNR or spike count. Compared to uncoated platinum, PEDOT‐CS showed an increase in geometric electrode area (125% larger than uncoated), linear diffusion electrode area (250% larger than uncoated), radial diffusion electrode area (207% larger than uncoated), decrease in total impedance at 1 kHz (12% of uncoated) and 12 Hz (1.6% of uncoated), **decrease** in RMS_bkgd_ (53% of uncoated), **increase** in SNR (190% larger than uncoated) and **increase** in spike count (248% larger than uncoated) (Harris et al. 2025). Both activated iridium and conducting polymers increase the charge storage capacity and charge density of the electrodes (Harris et al. 2017; Harris et al. 2016b).

Activated iridium and PEDOT‐CS modified electrodes had similar reductions in total impedance but radically different electrophysiological performance. The decrease in impedance associated with modifying an electrode with PEDOT‐CS is attributed to an increased electrode area and a new faradaic reaction. In contrast, the reduced impedance associated with iridium activation is solely attributed to the introduction of faradaic reactions. This implies that electrode area, but not faradaic reactions, impacts electrophysiological recordings.

Increased electrode area reduces thermal noise according to Equation (4). The biological noise also decreases due to changes in local potential associated with variations in ion concentration being averaged over a larger electrode‐tissue interface (Lempka et al. 2011). Larger electrodes also have more neurons within recording distance. Subsequently, increased electrode area reduces RMS_bkgd_ and increases SNR when recording population bursts. We note that changes in measured voltage associated with the firing of a single neuron would decrease with increased electrode area, but further modelling is required to determine the impact on SNR. Further modelling is also required to understand how more complex neural tissue structures and electrophysiological behaviour are impacted by electrode size.

While electrode size impacts electrophysiological performance, there are different measures of electrode area (Trasatti and Petrii 1992; Harris et al. 2018a). The geometric area, determined by microscopy may be sufficient for gauging electrophysiological performance of planar electrodes; however this can be difficult to measure on non‐planar electrode geometries, and does not account for surface roughness, electrodes with slow electron transfer kinetics, high resistivity or blocked surface regions. A more useful measure of effective electrode area can be obtained by performing cyclic voltammetry of a dissolved redox species with a high charge transfer rate, such as Ru(NH_3_)_6_^3+^ and use of appropriate electrochemical equations or modelling such as DigiElch (Harris et al. 2018a; Harris, Molino, et al. 2014; Harris et al. 2021; Harris 2021). Other methods for determining the electroactive electrode area, such as hydride adsorption or capacitance charge, can be inaccurate, dependant on experimental conditions and only available on specific electrode materials, limiting its utility for wide application (Harris et al. 2018a). The steady‐state electroactive area is a diffusion controlled measurement with a timescale far greater than an action potential. The linear diffusion electroactive area is obtained at shorter timescales (diffusion lengths). Correlations of electrode area and electrophysiological recordings with conducting polymer modified electrodes consistently showed stronger correlations with linear diffusion electroactive area (Harris et al. 2025). Further modelling is required to determine the relationship between diffusion length of different chemical species, electrode geometries and electrophysiological performance (this electrochemical detail is ignored in cable theory commonly used in electrophysiology modelling).

For impedance to be a relevant measurement for predicting electrophysiological performance, it must determine the effective electrode area. To achieve this, the measurement must be below the Maxwell‐Wagner cut‐off frequency, where impedance is dominated by the electrode double layer (the bulk solution properties dominate the behaviour above this frequency) (Harris et al. 2019). The measurement must be further analysed to determine the electrode area contribution without faradaic reaction contribution. This is typically achieved by fitting an equivalent circuit and isolating the capacitance or admittance parameter associated with the electrode area. However, there can be difficulties in this approach due to error in the fitting process. Furthermore, different electrode materials may require different equivalent circuits, preventing useful/simple comparison. Reporting of impedance at one frequency does not provide sufficient information for gauging the electrochemical or electrophysiological performance of an electrode; nor does a decrease in impedance infer improved electrophysiological performance.

Finally, this work demonstrates the presence (or introduction) of faradaic reactions into neural electrodes does not impact its recording performance. Iridium and iridium oxide can be used interchangeably in a combined neural stimulation and recording device. Changes in oxidation state due to neural stimulation should not impact the neural recordings. However, higher levels of oxidation may change its conductivity or corrode it sufficiently to impact on performance.

## 4 Conclusion

These results demonstrate that large changes in total impedance (at 1 kHz or 12 Hz), certain fitted equivalent circuit parameters and steady‐state electroactive area can have no detectable impact on electrophysiological recordings. The changes in impedance when activating iridium relate to the formation of a redox process at 0 V (where the EIS measurements were performed) and the associated appearance of a Warburg impedance. It can subsequently be inferred that incorporation of faradaic reactions into neural electrode coatings in order to reduce impedance will not improve neural recording performance.

Previous work with conducting polymer modified electrodes has shown that increasing the linear diffusion electroactive area and associated decrease in impedance (at low‐intermediate frequencies) resulted in a moderate reduction in RMS_bkgd_ but a large increase in the SNR and spike count difference (Harris et al. 2019; Harris et al. 2025). Together, these studies corroborate the finding that increased linear diffusion electroactive area has a moderate impact in reducing thermal noise and RMS_bkgd_ but substantially increases the population of neurons within recording distance of the electrode, resulting in an increased SNR and spike count. And despite a near order of magnitude variation in the steady‐state diffusion electroactive area of activated iridium, this measure had minimal impact on electrophysiological performance. The steady‐state diffusion electroactive area, which assesses longer time scale diffusion processes, is a poor measure of the population of neurons within recording distance. Therefore, the benefit of measuring electrode impedance of neural implants is largely related to its indirect assessment of linear diffusion electroactive area, in which case the measurement and analysis must be undertaken appropriately.

Finally, this work has demonstrated that a wide range of iridium activation states can be produced with increased charge injection capacity, but with no impact on the neural recording performance. This allows activated iridium to be used for both stimulating and recording electrodes, even with ongoing iridium oxidation occurring during neural stimulation, without fear of it impacting the recording experiments. However, higher oxidation states resulting in electrode dissolution or electrode resistance still limit the chronic performance of iridium oxide.

## Author Contributions

**Alexander R. Harris:** conceptualization, data curation, formal analysis, investigation, methodology, writing – original draft. **Ben J. Allitt:** investigation, writing – review and editing. **Antonio G. Paolini:** resources.

## Funding

Funding from the Australian Research Council Centre of Excellence Scheme (Project Numbers CE0561616 and CE140100012) is gratefully acknowledged.

## Conflicts of Interest

The authors declare no conflicts of interest.

## Supporting information


**Figure S1:** Reduction sweep obtained from cyclic voltammetry of 5 mM Ru(NH_3_)_6_^3+^ in 0.3 M Na_2_HPO_4_ after background subtraction at a voltammetric scan rate of 600 mV s^−1^ of iridium electrodes activated with 0, 50, 125, 250 or 500 pulses. Reproduced from Harris et al. (2017).
**Figure S2:** SNR comparison of iridium oxide (current manuscript) and PEDOT‐CS electrodes (reproduced from Harris et al. 2025) versus impedance at 12 Hz.

## Data Availability

Data are available for request from the author.
