# Supplementary material for: Electrophysiological Recording With Activated Iridium Oxide: Effective Electrode Area, Not Total Impedance, Determines Performance
Source: Eur J Neurosci. 2026 Jul 20;64(2):e70640. doi: 10.1111/ejn.70640 (PMC13385468; doi:10.1111/ejn.70640)
Supplement: Supplementary file 1 — Figure S1: Reduction sweep obtained from cyclic voltammetry of 5 mM Ru(NH3)6 3+ in 0.3 M Na2HPO4 after background subtraction at a voltammetric scan rate of 600 mV s−1 of iridium electrodes activated with 0, 50, 125, 250 or 500 pulses. Reproduced from Harris et al. (2017). Figure S2: SNR comparison of iridium oxide (current manuscript) and PEDOT‐CS electrodes (reproduced from Harris et al. 2025) versus impedance at 12 Hz. [file EJN-64-0-s001.docx]

Supporting Information

Electrophysiological Recording With Activated Iridium Oxide: Effective Electrode Area, not Total Impedance Determines Performance

Alexander R. Harris^1*^, Ben J. Allitt^2^, Antonio G. Paolini^3,4^

^1^ Department of Biomedical Engineering, University of Melbourne, Melbourne, VIC, 3010, Australia

^2^ Higher Education College, Chisholm Institute, Dandenong, Victoria, 3175, Australia

^3^ ISN Psychology, Institute for Social Neuroscience, Ivanhoe, Victoria 3087, Australia

^4^ School of Psychology and Public Health, La Trobe University, Bundoora, Victoria, 3086, Australia

*Email: alexrharris@gmail.com

Figure S1. Reduction sweep obtained from cyclic voltammetry of 5 mM Ru(NH_3_)_6_^3+^ in 0.3 M Na_2_HPO_4_ after background subtraction at a voltammetric scan rate of 600 mV s^-1^ of iridium electrodes activated with 0, 50, 125, 250 or 500 pulses. Reproduced from (Harris *et al.*, 2017).

Figure S2: SNR comparison of iridium oxide (current manuscript) and PEDOT-CS electrodes (reproduced from (Harris *et al.*, 2025)) versus impedance at 12 Hz.

**References**

Harris, A.R., Allitt, B., & Paolini, A.G. (2025) Electroanalysis and Electrophysiological Recording of Bio-Doped Conducting Polymer Modified Neural Electrodes. *Ann. N. Y. Acad. Sci.*, **1549**, 317–331.

Harris, A.R., Paolini, A.G., & Wallace, G.G. (2017) Effective Area and Charge Density of Iridium Oxide Neural Electrodes. *Electrochim. Acta*, **230**, 285–292.
